# The metabolic theory of ecology and the cost of parasitism

**DOI:** 10.1371/journal.pbio.2005628

**Published:** 2018-04-02

**Authors:** Mary I. O’Connor, Joanna R. Bernhardt

**Affiliations:** Department of Zoology and Biodiversity Research Centre, University of British Columbia, Vancouver, Canada

## Abstract

With over 1 million species on earth, each biologically unique, do we have any hope of understanding whether species will persist in a warming world? We might, because it turns out that there is surprising regularity in how warming accelerates the major metabolic processes that power life. A persistent challenge has been to understand ecological effects of temperature in the context of species interactions, especially when individuals not only experience temperature but also mortality due to parasitism or predation. Kirk et al. have shown how the effects of parasites vary with warming in a manner entirely consistent with general temperature dependence of host and parasite metabolism.

## The tyranny of temperature in ecological systems

Life at all scales—from *E*. *coli* to elephants—is powered by metabolism. Metabolic processes convert resources and energy to do the work of life. Warming temperatures accelerate metabolic rates by increasing the kinetic energy of biochemical systems. Despite the bewildering complexity of metabolism, two metabolic processes—aerobic respiration, oxygenic photosynthesis—are shared across diverse groups of animals, plants, and many microbes and fungi. The importance and constancy of these metabolic processes across the diversity of life lends some intriguing (and promising) predictability to how living systems respond to temperature change.

**Fig 1:**
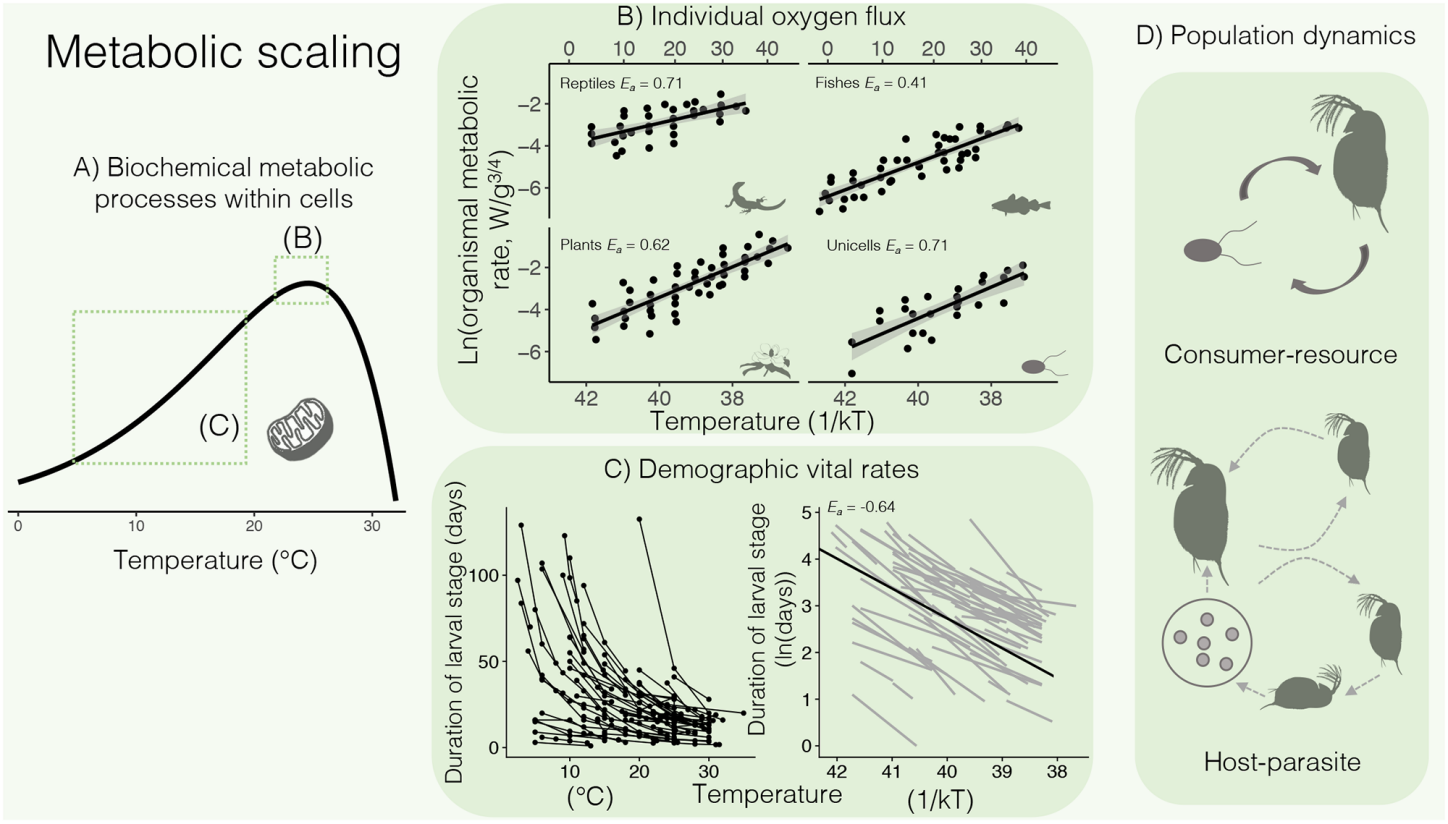
The MTE posits that temperature constrains rates of metabolic processes within cells, and these constraints emerge at higher levels of biological organization, such as individuals, populations, and species interactions. Within individuals, constraints imposed by temperature on cellular respiration and associated biological processes (A) can be estimated as the exponential increase of metabolic rate over a temperature gradient (described by the activation energy parameter *E*_*a*_). Very similar *E*_*a*_ values characterize the relationship between mass-normalized organismal respiration rate and temperature across species from a wide range of taxonomic diversity (B) (for ease of interpretation, lower horizontal axes are shown in reversed 1/kT, where k is Boltzmann’s constant and T is temperature in Kelvin, while upper horizontal axes are in °C; data from [2]). The temperature dependence of respiration constrains demographically important rates, such as development rate and its inverse, development time, which decreased exponentially with increasing temperature in 72 marine animals (C). The exponential effects of temperature shown in the left panel are often log transformed for analysis, allowing *E*_*a*_ to be described as a slope on an Arrenhius plot (right panel) (data from [9]). Temperature-dependent performance influences the outcomes of species interactions, including consumer–resource and host–parasite dynamics (D). *E*_*a*_, activation energy; MTE, metabolic theory of ecology.

This signal of temperature on the pace of life across ecological systems and species has been described in the metabolic theory of ecology (MTE) [1–5]. Remarkably, the effect of temperature on vital rates, such as growth or development, and on biological energy and material fluxes (e.g., respiration) is quantitatively consistent with how temperature affects key metabolic processes of respiration or photosynthesis at the subcellular level [2,6,7] (Fig 1). Consequently, the flux of energy and materials driven by metabolic processes increases predictably with warming at scales of cells [2], organisms [8,9], populations [4,10,11], communities [12,13], and ecosystems [3,14] and even along biogeographic gradients [7].

The inescapable effects of temperature on fundamental metabolic rates provide context and constraints for myriad adaptations of traits that minimize the otherwise extreme consequences of excessively cold or warm environments [15–17]. For example, within hundreds of species examined so far, development times of marine larvae, zooplankton, and fish increase exponentially as temperatures decline [8,9], but negative effects of slow development, such as increased vulnerability to predation or starvation, have been overcome by some species in cold climates through evolution of life histories that involve increased parental care of offspring [18]. Evolutionary processes have only somewhat modified the temperature dependence of photosynthesis or respiration across different life forms but instead have acted on other, more labile, traits to generate patterns in growth and resource-use traits across thermal gradients, which can compensate for the effects of temperature on metabolic performance [19,20]. In plants, adaptation of leaf and tree traits may erase the signal of temperature-dependent photosynthesis on canopy production at certain spatial and temporal scales, even though the underlying photosynthetic processes are still predictably sensitive to temperature [19]. Evolution plays out in the context of resource limitation, competition, facilitation, and selection by predation and parasitism, leading to complex ecological systems whose function and structure may not be clearly attributable to temperature variation alone. However, MTE offers an approach to understanding ecological systems that begins with the highly repeatable temperature dependence of metabolism and then considers how evolutionary and ecological processes play out, given this constraint imposed by temperature, providing a multi-scale framework for understanding how ecological systems change with temperature.

## Metabolic scaling and the outcomes of species interactions

One area in which the importance of temperature-dependent metabolism has been more difficult to understand is in the domain of population-level processes at local scales. This is because, while temperature-dependent metabolic rates affect vital rates of births and deaths directly, these vital rates are also affected by predation, disease, parasitism, resource supply, competition, or allocation. Metabolic scaling as a unifying principle that includes the dynamics of one or a few species has been more elusive, and complicated by evidence that general metabolic temperature dependence is potentially overwhelmed by the complexity, contingency, and context dependence of how temperature affects physiological traits, demographic processes, and their interactions [21–23]. Compounding the problem, empirical tests that actually measure demographic rates over temperature gradients under conditions that meet the assumptions of MTE are few and far between [24].

When the temperature dependence of fundamental metabolic rates is used in mathematical models to predict demographic vital rates and the outcomes of species interactions, the outcomes suggest nonintuitive shifts in abundance and persistence of populations with warming [10,25], predator–prey interactions [26], and host–parasite interactions [27]. These outcomes are not directly proportional to the effects of temperature on metabolism because population dynamics mediate the relationship between temperature, metabolism, and abundance. However, MTE and associated empirical tests [10,28] suggest that under simple conditions (minimal stress or mortality), temperature-dependent metabolism underlies processes at the population level in a manner consistent with models of general metabolic scaling. This is one of the most promising approaches ecological science has right now to understand how environmental temperature affects the dynamics and future persistence of ecological systems in a changing world.

## A new look at a host–parasite interaction in a warming world

Kirk et al. [29] combined mathematical and experimental approaches to determine whether metabolic scaling theory helped to understand how temperature affects the cost of parasitism. Parasites require a host for all or part of their life cycle, using energy or resources of their hosts and often costing the host its life or a portion of its ability to pass on genes to the next generations. All living organisms are vulnerable to parasites, and changes to host–parasite interactions can have devastating consequences for plant or animal populations. Changes to the cost of parasitism resulting from environmental change can be a critical component of the evolutionary trajectory of a population and even affect its ability to persist.

In their experiment, Kirk et al. [29] exposed healthy water fleas (*Daphnia magna*) to a microsporidian parasite (*Ordospora colligata*) at a range of 9 temperatures. They measured the effect of temperature on key attributes of the host–parasite species interaction. Importantly, their 9 temperature levels allowed them to estimate the functional form of the temperature dependence of these vital rates. Both *Daphnia* and parasites were affected by temperature; *Daphnia* life spans declined as temperatures increased over most of the temperature range, except for increases in life span with warming at the lowest temperatures [29], and parasites were able to infect *Daphnia* at higher rates at higher temperatures.

**Fig 2:**
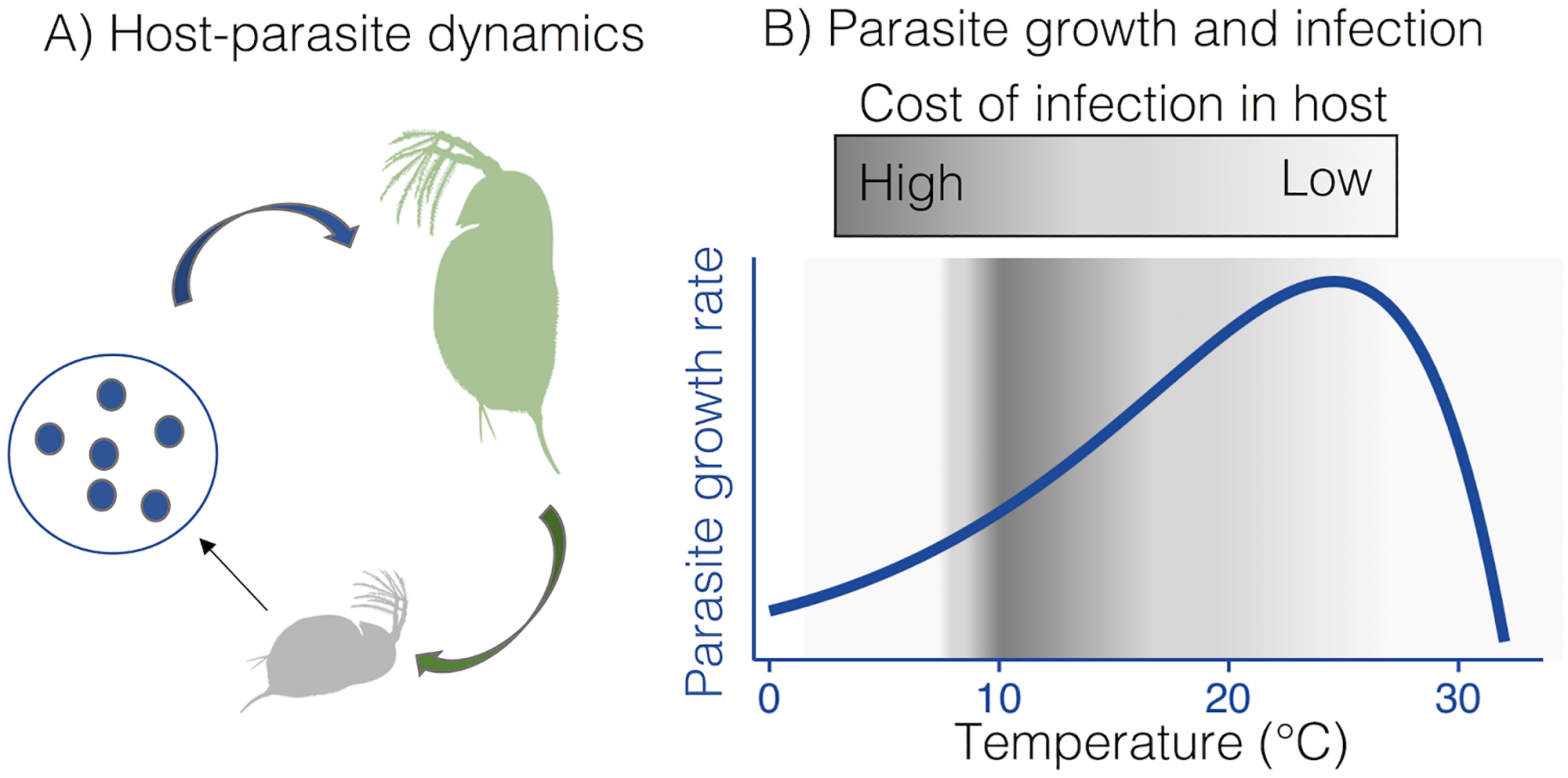
Metabolic scaling in the context of host–parasite dynamics. Using a combination of experiments and mathematical modeling, Kirk et al. [29] show that host survival, parasite growth, and the cost of infection in *Daphnia magna* can be predicted based on the temperature dependence of metabolic processes (A). Parasite growth rate increased with temperature, and the temperature dependence of parasite growth reflected the temperature dependence of cellular respiration, consistent with MTE. A mechanistic model of within-host parasite population dynamics, based on parameters derived from MTE (e.g., parasite growth rate), accurately predicted host life span, which was highest at intermediate temperatures (B). MTE, metabolic theory of ecology.

The effect of temperature on parameters that influence host–parasite dynamics, such as parasite population growth rate and *Daphnia* survival rate, were consistent with expectations for how temperature affects respiratory metabolism [2,4] and differed over the temperature gradient (Fig 2). Kirk et al. [29] compared 2 approaches to describing the effects of temperature on growth and mortality rates: first, they estimated these parameters at each temperature separately, and secondly, they fit a Sharpe-Schoolfield model with an Arrhenius function describing the increase in performance with temperature to all responses across all temperatures. They found that the model with the Arrhenius function, as predicted by MTE, was a more accurate and efficient description of how temperature affected *Daphia* and its parasite over the temperature gradient. Kirk et al. [29] then used this function to model population dynamics and estimate the cost of parasitism. The temperature dependences of other population attributes, abundance, and declines in life spans under physiologically stressful conditions were not well aligned with predictions based on the temperature dependence of respiration. It was expected that parasite abundance would be negatively related to the temperature dependence of respiration [4] and potentially modified by changes in parasite phenotype. For the curvature parameter that describes the decline in *Daphnia* performance at high temperatures, MTE does not predict a relationship between this and the temperature dependence of respiration.

Kirk and colleagues also report that the demographic consequences of temperature-dependent vital rates in this host–parasite interaction explain a maximal cost of infection at intermediate temperatures, even though population growth rates of the parasite are greatest at warmer temperatures (Fig 2). Such relationships between demographic outcomes and individual metabolic rates are no surprise to many population ecologists focusing on bioenergetics [30]. However, dynamic outcomes that reflect nonlinear changes to individual performance and population dynamics are not always considered when results of global change experiments are extended to predictions for effects of climate change. Kirk et al. [29] offer an important reminder that comparing experimental outcomes with metabolic theory predictions and their potential implications requires consideration of the dynamics that link temperature and an emergent response, such as abundance. When these researchers took this approach, they found that the metabolic theory of ecology can explain how temperature affects species interactions and their outcomes.

Kirk et al. [29] show that MTE leads to accurate predictions that can be applied over a continuous range of temperatures (in contrast to other models that can only be applied at discrete temperatures, for which empirical data are available). Their approach, based on theory, complements another leading framework for predicting global change impacts, the multiple stressor framework. Kirk and colleagues’ finding of a high cost of infection at intermediate temperatures but a small cost of infection at high and low temperatures might not have been predicted by the multiple stressor framework, which emphasizes responses to temperature at extreme temperatures. This finding highlights the importance of considering biological responses to temperature as continuous functions that reflect both stressful and nonstressful metabolic processes.

## Conclusions

The metabolic theory of ecology has provided new opportunities to understand how ecological systems grow and change across scales of space, time, and biological organization. This framework has challenged existing paradigms historically restricted to a narrower ecological scale but has also suggested that energetic constraints on metabolism are highly general and constrain demographic and evolutionary outcomes. Building on this, we are in a better position to project future ecological states from a theoretical framework with a broad conceptual and empirical domain, rather than making predictions by extrapolating data or models of restricted scope. Kirk et al. [29] demonstrate that MTE can be a powerful framework for predicting disease dynamics over gradients of temperature and for making predictions in the context of climate change.

## Abbreviations

*E_a_*: activation energy
MTE: metabolic theory of ecology
